# Mechanism of Follicular Helper T Cell Differentiation Regulated by Transcription Factors

**DOI:** 10.1155/2020/1826587

**Published:** 2020-07-20

**Authors:** Long-Shan Ji, Xue-Hua Sun, Xin Zhang, Zhen-Hua Zhou, Zhuo Yu, Xiao-Jun Zhu, Ling-Ying Huang, Miao Fang, Ya-Ting Gao, Man Li, Yue-Qiu Gao

**Affiliations:** ^1^Laboratory of Cellular Immunity, Institute of Clinical Immunology, Shanghai Key Laboratory of Traditional Chinese Clinical Medicine, Shuguang Hospital, Affiliated to Shanghai University of Traditional Chinese Medicine, Shanghai 201203, China; ^2^Department of Hepatopathy, Shuguang Hospital, Affiliated to Shanghai University of Traditional Chinese Medicine, Shanghai 201203, China

## Abstract

Helping B cells and antibody responses is a major function of CD4^+^T helper cells. Follicular helper T (Tfh) cells are identified as a subset of CD4^+^T helper cells, which is specialized in helping B cells in the germinal center reaction. Tfh cells express high levels of CXCR5, PD-1, IL-21, and other characteristic markers. Accumulating evidence has demonstrated that the dysregulation of Tfh cells is involved in infectious, inflammatory, and autoimmune diseases, including lymphocytic choriomeningitis virus (LCMV) infection, inflammatory bowel disease (IBD), systemic lupus erythematosus (SLE), rheumatoid arthritis (RA), IgG4-related disease (IgG4-RD), Sjögren syndrome (SS), and type 1 diabetes (T1D). Activation of subset-specific transcription factors is the essential step for Tfh cell differentiation. The differentiation of Tfh cells is regulated by a complicated network of transcription factors, including positive factors (Bcl6, ATF-3, Batf, IRF4, c-Maf, and so on) and negative factors (Blimp-1, STAT5, IRF8, Bach2, and so on). The current knowledge underlying the molecular mechanisms of Tfh cell differentiation at the transcriptional level is summarized in this paper, which will provide many perspectives to explore the pathogenesis and treatment of the relevant immune diseases.

## 1. Introduction

CD4^+^helper T cells play a critical role in forming and amplifying the abilities of the immune system. Follicular helper T (Tfh) cells are identified as a subset of CD4^+^T helper cells, which provides help to B cells for the formation and maintenance of the germinal center (GC) , the production of high affinity class-switched antibodies, long-lived plasma cells, and memory B cells [1]. There were a great deal of researches about Tfh cells in the past 10 years; in particular, the differentiation and function of Tfh cells were involved in a range of diseases including infectious diseases, vaccines, autoimmune diseases, and allergies. Tfh cells are characterized by high expression of the chemokine receptor CXCR5, the transcription factor Bcl6, the costimulatory molecule ICOS, and the coinhibitory molecule PD-1. Once naïve CD4^+^T cells are activated by antigen-presenting cells (APCs) together with IL-6 and IL-21, they will differentiate into Tfh cells.

**Figure 1:**
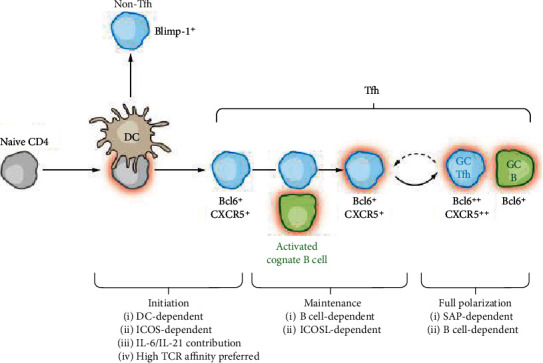
The differentiation of Tfh cells: multiple stages of Tfh cell differentiation, including initiation, maintenance, and full polarization stages (Annual Review of Immunology, 2011,29(1):621-663).

A multiple-stage process is involved in the generation of Tfh cells from naïve CD4^+^T cells, which consists of initiation, maintenance, and full polarization stages [1]. During the initiation phase of Tfh cell differentiation, multiple signals take part in the process, including transcription factors (Bcl6, Ascl2, Batf, IRF4, c-Maf, and so on), costimulatory molecule(ICOS), and cytokines(IL-6/IL-21); in particular, higher TCR affinity is necessary for initiation of Tfh cell (Bcl6^+^CXCR5^+^) differentiation at the phase of dendritic cell priming [2–7]. Then, Bcl6^+^CXCR5^+^ Tfh precursor cells move into the T-B border zone, where they accept other differentiation signals from activated B cells [8]. After this appointment, the toughened expression of Bcl6 regulates surface markers, which accelerates the migration of Tfh cells into GC, where they offer assistant signals for B cells [9, 10] (Figure 1).

**Figure 2:**
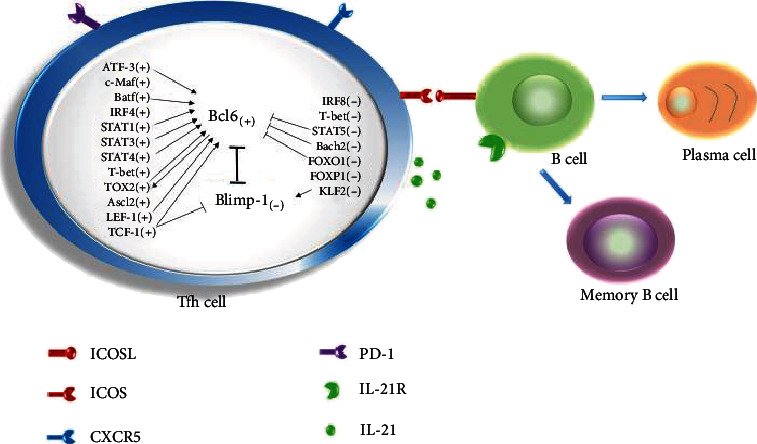
Network of transcription factors in the differentiation of Tfh cells. Tfh cells are regulated by a complex network of transcription factors, including Bcl6, Blimp-1, ATF-3, c-Maf, Batf, IRF4, IRF8, STATs, T-bet, TOX2, Ascl2, LEF-1, TCF-1, Bach2, FOXO1, FOXP1, and KLF2. “+” means positive factors and “−” means negative factors. “→” means the promoting effect and “⊣” means the inhibitory effect.

Differentiation of naïve CD4^+^T cells into Tfh cells is modulated by a multipart transcriptional network (Figure 2). Multiple transcription factors that either support or oppose the differentiation and function of Tfh cells have been identified (Table 1).

Now, the knowledge of the transcriptional mechanism underlying Tfh cell differentiation will be comprehensively described in this paper, which will highlight the possible future directions.

## 2. Bcl6 and Blimp-1

Bcl6 has been known as a key transcription factor for Tfh cell development by pathways essentially independent of Blimp-1 [3, 39]. Bcl6 consists of a zinc finger domain; a bric-a-brac, tramtrack, broad-complex (BTB) domain; and a middle domain [40]. The Bcl6 DNA binding zinc finger domain is essential for Bcl6 activity in CD4^+^T cells [8]. The BTB domain of Bcl6 participates in the correct differentiation of Tfh cells most likely by interacting with Bcl6-interacting corepressor (BCOR) [41]. The middle domain of Bcl6 prevents the association with the corepressor metastasis-associated protein 3 (MTA3) and inhibits the differentiation and function of Tfh cells by distressing Prdm1 (encodes Blimp-1) and other crucial target genes [42].

Bcl6 expression is induced by IL-6-STAT1/STAT3 signaling [43], and it is driven very early after T cell activation in a CD28-dependent manner [44]. The E3 ubiquitin ligase Itch is essential for Bcl6 expression at the early stage of Tfh cell development [45]. The deficiency of the Wiskott-Aldrich syndrome protein suppresses Bcl6 transcription, which results in a deficient response of Tfh cells [46]. Research shows that Bcl6 inhibits the IL-7R/STAT5 axis during Tfh cell generation [47]. Bcl6 mediates the effect of activating transcription factor 3 (ATF-3) on Tfh cells in the gut [16]. ATF-3 is a stress-inducible transcription factor and plays a critical role in the prevention of colitis by regulating the development of Tfh cells in the gut. In addition, Bcl6 also suppresses the expression of specific microRNAs that are thought to control the differentiation of Tfh cells, such as miR-17-92 [9] and miR-31 [48]. The miR-17-92 inhibits CXCR5 expression, and miR-31 directly binds to Bcl6 promoter.

Blimp-1 has been found to be a critical transcriptional repressor for Tfh cell differentiation. Blimp-1 has the inhibitory effect on Bcl6 expression, indicating that Bcl6 and Blimp-1 are antagonistic regulators in the differentiation of Tfh cells. Blimp-1 is induced by IL-2/STAT5 signaling, and it suppresses the expression of Tfh-associated genes including Bcl6, c-Maf, Batf, CXCR5, and IL-21 [25, 26]. Blimp-1-deficient CD4^+^T cells in mice show enhanced Tfh cell differentiation and GC formation [3, 49]. Taken together, these results indicate that Bcl6 is both necessary and sufficient for Tfh cell development and the proper differentiation of Tfh cells in vivo, and the differentiation of Tfh cells requires keeping the expression balance between Bcl6 and Blimp-1.

Bcl6 and Blimp-1 are associated with various infectious and autoimmune diseases by regulating Tfh cells. Bcl6 is highly expressed in sinus tissues, parotid gland tissues, and lacrimal gland tissues of IgG4-related disease (IgG4-RD) patients [14]. Blimp-1 in peripheral blood is upregulated in patients with IgG4-RD [14]. Compared with the healthy controls, higher expression of Bcl6 and lower Blimp-1 expression in the peripheral blood are observed in patients with rheumatoid arthritis (RA) [15].

## 3. c-Maf and Batf

c-Maf and Batf are the members of the activator protein 1 (AP-1) family. c-Maf is a bZIP transcriptional factor , and promotes the differentiation of Tfh cells. [6]. It is highly expressed in Th17 cells and mature Tfh cells. The selective loss of c-Maf expression in Tfh cells results in the downregulated expression of Bcl6, CXCR5, PD-1, and IL-21 [6]. In addition, one study reveals that Bcl6 and c-Maf synergistically orchestrate the expression of Tfh cell-associated genes (PD-1, ICOS, CXCR5, and so on) [4].

Batf is known to control switched antibody responses. Batf is highly expressed in Tfh cells and is essential for the differentiation of Tfh cells through regulating the expression of Bcl6 and c-Maf [50, 51]. Batf directly binds to and activates the conserved noncoding sequence 2 (CNS2) region in the IL-4 locus and then triggers the production of IL-4 in Tfh cells [52].

Both c-Maf and Batf are related with immune diseases. Compared with the healthy controls, c-Maf mRNA expression level and percentage of Tfh cells in peripheral blood mononuclear cells (PBMCs) are increased in patients with chronic immune thrombocytopenia (cITP), and they are decreased after the effective treatment [12]. Compared with the healthy controls, Batf in the submandibular glands and affected lymph nodes is markedly increased in patients with IgG4-RD [17].

## 4. IRF4 and IRF8

IRF4 and IRF8 belong to the evolutionarily conserved IRF family. IRF4 is expressed in hematopoietic cells and plays pivotal roles in the immune response. It has been acknowledged that the IRF4 locus “senses” the intensity of TCR signaling to determine the expression level of IRF4 [18]. IRF4 plays a critical role in regulating the generation of Tfh cells. In IRF4^−/−^ mice, CD4^+^T cells in lymph nodes and Peyer's patches fail to express Bcl6 and other Tfh-related molecules [53]. IL-21 is a key cytokine for the development of Tfh cells [54], and IRF4 regulates the production of IL-21 [55]. Therefore, IL-21 takes part in regulating the differentiation of Tfh cells by IRF4. In wild-type mice, IRF4 can interact with Batf-JUN family protein complexes to form a heterotrimer that can bind to AP1-IRF4 complexes and regulate Tfh cell differentiation [50, 51].

IRF8 plays various and important regulatory roles in the growth, differentiation, and function of immune cells in inflammatory bowel disease (IBD) patients [19]. IRF8 inhibits the differentiation of Tfh cells by directly binding to the promoter region of the IRF4 gene and inhibiting the transcription and activation of IRF4. In contrast, IRF8 deficiency significantly enhances IRF4 binding the promoter region of the IL-21 gene and results in the expansion of Tfh cell differentiation in vitro and in vivo [19].

## 5. STATs

Members of the STAT family including STAT1, STAT3, STAT4, and STAT5 are the important regulators for the generation of Tfh cells [43, 54]. STAT1 is necessary for IL-6-mediated Bcl6 induction during the early differentiation of Tfh cells [43]. STAT3 has been found to be critical for Tfh cell development in a Bcl6-dependent manner [23]. The major STAT3-stimulating cytokines include IL-6, IL-21, IL-12, IL-10, and TGF-*β* [23, 56, 57]. Besides, STAT3 regulates Bcl6 expression by cooperating with the Ikaros zinc finger transcription factors Aiolos and Ikaros [58]. TRAF6 inhibits the activation of type I interferon-STAT3 signaling [59]. The latest research clearly shows that T-bet, although mildly inhibiting early Tfh cell differentiation, mainly plays a crucial and specific supporting role for Tfh cell response by promoting cell proliferation and apoptotic intervention at the end-stage effector phase of acute viral challenge [2]. T-bet and STAT4 are coexpressed with Bcl6 to coordinate the production of IL-21 and IFN-*γ* by Tfh cells and promote the GC response [24]. STAT5 is shown as an inhibitory factor for the differentiation of Tfh cells. Molecular analyses reveal that the activation of the IL-2/STAT5 signaling enhances the expression of Blimp-1 and prevents the binding of STAT3 to the Bcl6 locus [25], resulting in the decrease of GC and the long-lived antibody responses [26]. Similarly, IL-7-dependent activation of STAT5 contributes to Bcl6 repression [60]. The latest research shows that IL-10 suppresses the differentiation of Tfh cells in human and mice by promoting STAT5 phosphorylation [61]. He et al. [62] demonstrate that the secreted protein extracellular matrix protein 1 induced by IL-6 and IL-21 in Tfh cells promotes the differentiation of Tfh cells by downregulating the level of STAT5 phosphorylation and upregulating Bcl6 expression.

T-bet and STATs are the important regulators for Tfh cell development in infectious and autoimmune diseases. STAT1 serine-727 phosphorylation (designated STAT1-pS727) plays an important role in promoting Tfh cell responses, leading to systemic lupus erythematosus- (SLE-) associated autoantibody production [21]. Compared with the healthy controls, the expression levels of pSTAT1, pSTAT4, and T-bet in PBMCs are upregulated in patients with SLE [22]. The expression level of pSTAT3 in PBMCs in patients with RA is higher than that in healthy controls [23].

## 6. TCF-1 and LEF-1

TCF-1 is expressed in both developing and mature T cells and is essential for initiating and securing the differentiation of Tfh cells [7, 63]. TCF-1 directly binds to the Bcl6 transcription start site and Prdm1 5′ regulatory regions, which promotes the expression of Bcl6 and represses the expression of Blimp-1 during acute viral infection [7, 28, 29]. TCF-1 synergistically works with LEF-1 to promote the early differentiation of Tfh cells by the multipronged approach of maintaining the expression of IL-6Ra and gp130, enhancing the expression of ICOS, and promoting the expression of Bcl6 [30].

## 7. TOX2

The high-mobility group- (HMG-) box transcription factor TOX2 is selectively expressed in human Tfh cells and regulated by Bcl6 and STAT3 in the initial stage of Tfh cell generation [31]. There is a feed-forward loop centering on TOX2 and Bcl6, which drives Tfh cell development. TOX2 promotes Bcl6 expression by inhibiting IL-2 and/or enhancing IL-6 signaling during Tfh cell development. Furthermore, TOX2 is bound to the sites shared by Batf and IRF4, which suggests that TOX2, Batf, and IRF4 may functionally converge in developing Tfh cells.

## 8. Ascl2

Ascl2, a basic helix-loop-helix domain-containing transcription factor, is highly expressed in Tfh cells, and its expression may precede Bcl6 expression. The expression of Ascl2 in the spleen is upregulated in sjögren syndrome (SS) model mice compared with control mice [32]. Ascl2 initiates the differentiation of Tfh cells via upregulating CXCR5 and downregulating C-C chemokine receptor 7(CCR7) expression as well as the IL-2 level in T cells in vitro. The Ikappa BNS is highly expressed in Tfh cells and is essential for Ascl2-induced CXCR5 expression during the differentiation of Tfh cells [64]. After activation of the signals related to Tfh cells described above, Ascl2 accelerates T cell migration into the follicles in mice [5]. Acute deletion of Ascl2, as well as inhibition of its function with the Id3 protein, can result in impaired Tfh cell development and GC response [5]. In addition, epigenetic regulations, such as histone modifications, also coordinately control the differentiation and function of Tfh cells along with transcription factors. The Ascl2 locus is marked with the active chromatin marker trimethylated histone H3 lysine 4 (H3K4me3) in Tfh cells, and other transcription factors including Bcl6, Maf, Batf, and IRF4 are uniformly associated with H3K4me3 [65].

## 9. Bach2

Bach2 is a negative regulator of Tfh cell differentiation. Bach2 directly represses the expression of Bcl6 by inhibiting Bcl6 promoter activity [34] and negatively regulates CXCR5 expression [34]. Overexpression of Bach2 in Tfh cells inhibits the expression of Bcl6, IL-21, and the coinhibitory receptor TIGIT [34]. The deletion of Bach2 leads to the upregulation of CXCR5 expression and contributes to preferential Tfh cell differentiation [33].

## 10. FOXO1 and FOXP1

FOXO1 has been found to negatively regulate the differentiation of Tfh cells through the ICOS-mTORC2-FOXO1 signaling in the early stage of differentiation [66]. FOXO1 regulates the differentiation of Tfh cells by negatively regulating Bcl6. The E3 ubiquitin ligase Itch is essential for the differentiation of Tfh cells. Itch associates with FOXO1 and promotes its ubiquitination and degradation [45] and then positively regulates the differentiation of Tfh cells. FOXP1 negatively regulates the expression of CTLA-4 and IL-21 in activated CD4^+^T cells [36]. Naïve CD4^+^T cells deficient in the FOXP1 preferentially differentiate into Tfh cells, which results in substantially enhanced GC and antibody responses [67]. In addition, FOXP1-deficient Tfh cells restore the generation of high-affinity Abs when cocultured with high numbers of single clone B cells [36].

## 11. KLF2

The transcription factor KLF2 serves to inhibit Tfh cell generation by downregulating sphingosine-1-phosphate receptor (S1PR1). KLF2 deficiency in activated CD4^+^T cells contributes to Tfh cell generation, whereas KLF2 overexpression prevents Tfh cell production. KLF2 also induces the expression of Blimp-1 and thereby inhibits the differentiation of Tfh cells [38]. ICOS maintains the phenotype of Tfh cells by downregulating KLF2. KLF2 is identified as a target of miRNA92a in inducting the expression of the human Tfh precursor, and the miRNA92a-mediated Tfh precursor induction is regulated by PTEN-PI3K-KLF2 signaling [37].

## 12. Conclusions

Multiple transcription factors have been found to regulate Tfh cell generation. In this paper, the regulatory mechanisms of transcription factors on Tfh cell differentiation are summarized. However, many questions remain to be further investigated. (i) Are there other Tfh-specific transcription factors beyond the abovementioned factors? (ii) How do Tfh-specific transcriptional factors impact epigenetic mechanisms during inducing Tfh cell generation? (iii) What are the factors' stage-specific requirements? (iv) What are the molecular mechanisms contributing to Tfh cell maintenance and memory formation?

As summarized in this review, Tfh cell-related transcription factors including Bcl6, IRF4, STAT1/STAT4/STAT5, T-bet, TCF-1, LEF-1, TOX2, Bach2, FOXP1, and KLF2 are all involved in the virus infection. Both Bcl6 and STAT3 play an important role in RA. ="The expression levels of Bcl6, STAT1, STAT4 and T-bet are upregulated in SLE patients. Bcl6, Blimp-1, and Batf are associated with IgG4-RD. Due to the association of Tfh cells with a broad spectrum of diseases, subsequent in-depth investigation of regulatory factors for the differentiation of Tfh cells may provide the potential therapeutic targets for various immune diseases, especially the virus infection, SLE, RA, and IgG4-RD.

## Figures and Tables

**Table 1:** Transcription factors in the differentiation of Tfh cells.

Transcription factors	Abbreviation of transcription factors	Function in Tfh cell differentiation	Signaling pathways	Related diseases
B-cell lymphoma 6 protein	Bcl6	Initiates the differentiation of Tfh cells at the early stage of Tfh cell generation	Be induced by IL-6-STAT1/STAT3 signaling, inhibits the IL-7R/STAT5 axis, suppresses microRNAs (miR-17-92 and miR-31), a direct target of ATF-3	Acute lymphocytic choriomeningitis virus (LCMV) infection [11]
				Chronic immune thrombocytopenia (cITP) [12]
				Systemic lupus erythematosus (SLE) [13]
				IgG4-related disease (IgG4-RD) [14]
				Rheumatoid arthritis (RA) [15]
B lymphocyte maturation protein 1	Blimp-1	Inhibits the differentiation of Tfh cells	Inhibits the expression of Bcl6	IgG4-RD [14]
Activating transcription factor 3	ATF-3	Initiates the differentiation of Tfh cells	Targets Bcl6 in CD4^+^T cells	Colitis [16]
c-Musculoaponeurotic-fibrosarcoma	c-Maf	Promotes the differentiation of Tfh cells	c-Maf and Bcl6 synergistically orchestrate genes that define core characteristics of Tfh cell biology	cITP [12]
Basic leucine zipper transcription factor	Batf	Promotes the differentiation of Tfh cells	Regulates the expression of Bcl6 and c-Maf, cooperates with IRF4 during the development of Tfh cells, be regulated by IL-4-STAT6 and IL-6-STAT3 signaling	IgG4-RD [17]
Interferon regulatory factor 4	IRF4	Promotes the differentiation of Tfh cells	Regulates the generation of Tfh cells in a Bcl6-dependent manner, regulates the production of IL-21 and controls most IL-21-regulated genes by IL-21-STAT3 axis	LCMV infection [18]
Interferon regulatory factor 8	IRF8	Inhibits the differentiation of Tfh cells	Binds to the promoter region of IRF4 gene and inhibits transcription and transactivation of IRF4	Inflammatory bowel disease (IBD) [19]
Signal transducers and activators of transcription 1	STAT1	Promotes the differentiation of Tfh cells at the early stage of Tfh cell generation	Regulates the differentiation of Tfh cells by IL-6-STAT1-Bcl6 signaling	Viral infection [20]
				SLE [21, 22]
Signal transducers and activators of transcription 3	STAT3	Promotes the differentiation of Tfh cells	Regulates the expression of Bcl6	RA [23]
Signal transducers and activators of transcription 4	STAT4	Promotes the differentiation of Tfh cells	STAT4 and T-bet are coexpressed with Bcl6 to coordinate the production of IL-21 and IFN-*γ* by Tfh cells	Acute LCMV infection [24]
				SLE [22]
Signal transducers and activators of transcription 5	STAT5	Inhibits the differentiation of Tfh cells	Downregulates Bcl6 expression and upregulates Blimp-1 expression through IL-2/IL-7/IL-10-STAT5 signaling	LCMV infection [25, 26]
T-box expressed in T cells	T-bet	Mildly inhibits the early differentiation of Tfh cells, but promotes Tfh cell proliferation and apoptotic intervention at the late effector phase	T-bet and STAT4 are coexpressed with Bcl6 to coordinate the production of IL-21 and IFN-*γ* by Tfh cells	LCMV infection [2, 24, 27]
				SLE [22]
T cell-specific transcription factor 1	TCF-1	Promotes the differentiation of Tfh cells at the early stage of Tfh cell generation	Promotes the expression of Bcl6, but represses the expression of Blimp-1	LCMV infection [7, 28–30]
Lymphoid enhancer binding factor 1	LEF-1	Promotes the differentiation of Tfh cells at the early stage of Tfh cell generation	Works synergistically with TCF-1 to enhance the expression of ICOS and Bcl6	LCMV infection [30]
The high-mobility group- (HMG-) box 2	TOX2	Initiates the differentiation of Tfh cells	Be regulated by Bcl6 and STAT3 in the initial stage of Tfh cell generation, inhibits IL-2 and/or enhances IL-6 signaling to promote Bcl6 expression	Viral infection [31]
Achaete-scute homolog 2	Ascl2	Promotes the differentiation of Tfh cells	Upregulates CXCR5 but not Bcl6 and downregulates CCR7 expression as well as IL-2 signaling	Sjögren syndrome (SS) [32]
BTB and CNC homolog 2	Bach2	Inhibits the differentiation of Tfh cells	Suppresses the expression of Bcl6 by directly binding to the promoter, negatively regulates CXCR5 expression	Viral infection [33, 34]
Forkhead-box protein O1	FOXO1	Inhibits the differentiation of Tfh cells	Negatively regulates the differentiation of Tfh cells through an ICOS-mTORC2-FOXO1 signaling axis in the early stages of differentiation, negatively regulates the expression of Bcl6	Angioimmunoblastic T cell lymphoma induced [35]
Forkhead-box protein P1	FOXP1	Inhibits the differentiation of Tfh cells	Negatively regulates the expression of CTLA-4 and IL-21 in activated CD4^+^T cells	LCMV infection [36]
Krüppel-like factor 2	KLF2	Inhibits the differentiation of Tfh cells	Downregulates S1PR1, induces the expression of Blimp-1, miRNA92a-mediated Tfh precursor induction is regulated by PTEN-PI3K-KLF2 signaling	Type 1 diabetes (T1D) [37]
				LCMV infection [38]
